# In-Home Monitoring Technology for Aging in Place: Scoping Review

**DOI:** 10.2196/39005

**Published:** 2022-09-01

**Authors:** Daejin Kim, Hongyi Bian, Carl K Chang, Liang Dong, Jennifer Margrett

**Affiliations:** 1 Department of Interior Design Iowa State University Ames, IA United States; 2 Department of Computer Science Iowa State University Ames, IA United States; 3 Department of Electrical and Computer Engineering Iowa State University Ames, IA United States; 4 Department of Human Development and Family Studies Iowa State University Ames, IA United States

**Keywords:** in-home monitoring, aging in place, ambient assisted living, home modification, monitoring, aging, technology, intervention, older adult, wellness, independence, monitor, research, sensor, activity, behavior, cognitive, sleep

## Abstract

**Background:**

For successful aging-in-place strategy development, in-home monitoring technology is necessary as a new home modification strategy. Monitoring an older adult’s daily physical activity at home can positively impact their health and well-being by providing valuable information about functional, cognitive, and social health status. However, it is questionable how these in-home monitoring technologies have changed the traditional residential environment. A comprehensive review of existing research findings should be utilized to characterize recent relative technologies and to inform design considerations.

**Objective:**

The main purpose of this study was to classify recent smart home technologies that monitor older adults’ health and to architecturally describe these technologies as they are used in older adults’ homes.

**Methods:**

The scoping review method was employed to identify key characteristics of in-home monitoring technologies for older adults. In June 2021, four databases, including Web of Science, IEEE Xplore, ACM Digital Library, and Scopus, were searched for peer-reviewed articles pertaining to smart home technologies used to monitor older adults’ health in their homes. We used two search strings to retrieve articles: types of technology and types of users. For the title, abstract, and full-text screening, the inclusion criteria were original and peer-reviewed research written in English, and research on monitoring, detecting, recognizing, analyzing, or tracking human physical, emotional, and social behavior. The exclusion criteria included theoretical, conceptual, or review papers; studies on wearable systems; and qualitative research.

**Results:**

This scoping review identified 30 studies published between June 2016 and 2021 providing overviews of in-home monitoring technologies, including (1) features of smart home technologies and (2) sensor locations and sensor data. First, we found six functions of in-home monitoring technology among the reviewed papers: daily activities, abnormal behaviors, cognitive impairment, falls, indoor person positioning, and sleep quality. Most of the research (n=27 articles) focused on functional monitoring and analysis, such as activities of daily living, instrumental activities of daily living, or falls among older adults; a few studies (n=3) covered social interaction monitoring. Second, this scoping review also found 16 types of sensor technologies. The most common data types encountered were passive infrared motion sensors (n=21) and contact sensors (n=19), which were used to monitor human behaviors such as bodily presence and time spent on activities. Specific locations for each sensor were also identified.

**Conclusions:**

This wide-ranging synthesis demonstrates that in-home monitoring technologies within older adults’ homes play an essential role in aging in place, in that the technology monitors older adults’ daily activities and identifies various health-related issues. This research provides a key summarization of in-home monitoring technologies that can be applied in senior housing for successful aging in place. These findings will be significant when developing home modification strategies or new senior housing.

## Introduction

### Background

As all of the “baby boomer” generation approaches exceeding the age of 65 years, by 2030, they will proportionally expand the older population in the United States, thus becoming a super-aged society in which older individuals will comprise more than 20% of the population. The US Census Bureau data project that by 2060, the 65-and-older population will total 98 million [1]. A recent American Association of Retired Persons survey in the United States indicated that 79% of older adults want to remain in their current home and community as they age [2]. The desire of older individuals to independently and safely remain in their homes and communities is referred to as *aging in place*. Successful aging in place supports the positive experience of an individual’s identity in that it helps to improve their independence and autonomy in their homes and community. Additionally, aging in place positively impacts older residents’ health and well-being. According to the Center for Housing Policy [3], approximately 80% of seniors have at least one chronic health condition and 50% have at least two. Many older adults with functional or cognitive decline have difficulties in living independently when performing basic daily activities in their homes. These challenges may reduce successful aging in place because their homes might fail to support their changing needs and gradually become dangerous environments for independent living.

*Home modification* positively affects older adults’ ability to age in place because it renders their homes safer and more accessible to both themselves and visitors, thus improving comfort and reducing accidents such as falling [4]. Home modifications, considered a promising tool for aging in place, are most beneficial when they provide tailored interventions for older adults that take into account specific health conditions such as mobility, cognitive impairment, and eye impairment, among others [5]. Thus, to support changing needs, it is important to understand how older adults interact with their residential environments.

*Smart home technologies* have been considered essential interventions that enable older adults to maintain wellness and independence at home [6,7]. Smart home technologies, representing a variety of sensors and devices integrated into the home infrastructure, provide a wide range of features for enhancing quality of life, from simple home automation to monitoring wellness [6]. Home automation provides remote or automatic controls for devices, appliances, or home systems that enhance an occupant’s quality of life in terms of entertainment and energy-saving. In-home monitoring can be employed to monitor an occupant’s health status and maintain a sense of well-being.

In particular, monitoring an older adult’s daily physical activity at home can provide valuable information about their functional, cognitive, and social health status; such monitoring information can indicate the individual’s ability to maintain function and independence at home [6]. With technological advancements proceeding at an unprecedented rate, smart home technologies such as the Internet of Things and ambient intelligence have become more intertwined with the fabric of everyday life and the environment. Technologies that monitor older adults’ health are easily accessible at affordable prices and constitute an active research area. For the development of a successful aging-in-place strategy, in-home monitoring technology is necessary as a new home modification strategy, which can positively impact older adults’ health and well-being. However, there is a huge knowledge gap between what in-home monitoring technologies are currently available and how these technologies can be incorporated into older adults’ homes. Although many review papers have described existing smart home monitoring technologies for older adults, they focused on the technological perspectives of in-home monitoring technologies, such as systems, networks, and computational frameworks. There are limited resources available that architects and designers can use for developing a smart home for successful aging in place. Moreover, it is questionable how these smart technologies have changed the traditional residential environment. Thus, to ultimately enable older adults to achieve successful aging in place, a comprehensive review of existing research findings is needed to characterize recent relative technologies and to inform design considerations.

### Aim of the Study

The main purpose of this study was to classify recent smart home technologies that monitor older adults’ health and to architecturally describe these technologies as they are used in older adults’ homes. The following research questions served as a guide for the study: (1) What in-home monitoring technologies are currently available for older adults? (2) Where can in-home monitoring technologies be installed and why are they used?

The findings of this research will provide empirical evidence of smart technology’s impact on aging in place, and help designers transform and tailor older adults’ living environments appropriately for the implementation of various technologies. This research will benefit various aging-in-place stakeholders, including older adults, family caregivers, clinicians, designers, public health practitioners, and policymakers. 

## Methods

### Overview

The scoping review method was employed to identify key characteristics of in-home monitoring technologies for older adults. This method can be used to summarize underexplored research areas and determine the nature of specific topics, identifying research gaps that remain after previous studies. This study was developed according to the methodological guideline for scoping reviews [7]: identifying relevant studies; study selection; charting the data; and collating, summarizing, and reporting results.

### Identifying Relevant Studies

In June 2021, four databases, including the Web of Science, IEEE Xplore, ACM Digital Library, and Scopus, were searched for peer-reviewed articles pertaining to smart home technologies used to monitor older adults’ health in their homes. The following keywords were applied when searching: (“Smart home” OR “gerontechnology” OR “monitoring” OR “ambient assisted living” OR “unobtrusive sensors” OR “in-home monitoring”) and (“Older adults” OR “Aging” OR “Ageing” OR “Elderly” OR “Senior” OR “Aging in Place”). To ensure the retrieved articles were up to date, the publishing span was limited to articles bearing dates between June 2016 and 2021.

### Study Selection

This scoping review was performed according to the PRISMA-ScR (Preferred Reporting Items for Systematic Reviews and Meta-Analyses extension for Scoping Reviews) guidelines [7,8], and involved a wide-ranging literature search for information on in-home monitoring technologies for older adults, employing multiple search strategies. For the title, abstract, and full-text screening, the inclusion and exclusion criteria were developed as shown in Textbox 1. This review excluded wearable sensors even though they are an important part of the monitoring strategy for older adults. This exclusion criterion was established owing to the usability barriers of these devices. For example, older adults with cognitive impairment may have difficulty in using a wearable sensor because these devices must be worn on a regular basis.

**Inclusion and exclusion criteria.**

**Inclusion criteria**

- Original and peer-reviewed research
- Paper written in English
- Research on monitoring, detecting, recognizing, analyzing, or tracking human physical, emotional, and social behavior
- Research with human subjects

**Exclusion criteria**

- Theoretical, conceptual, or review papers without empirical data or demonstrations
- Wearable systems
- Qualitative research

### Charting the Data

In response to the two research questions, (1) types of in-home monitoring technologies for older adults and (2) specifications of the sensors (eg, location and data), deductive thematic analysis was employed. Two authors (DK and HB) extracted data from the identified articles using an Excel file. The form included the following six factors: (1) purpose, (2) sample, (3) settings, (4) technologies, (5) technology type, and (6) outcome.

## Results

### Overview

**Figure 1:**
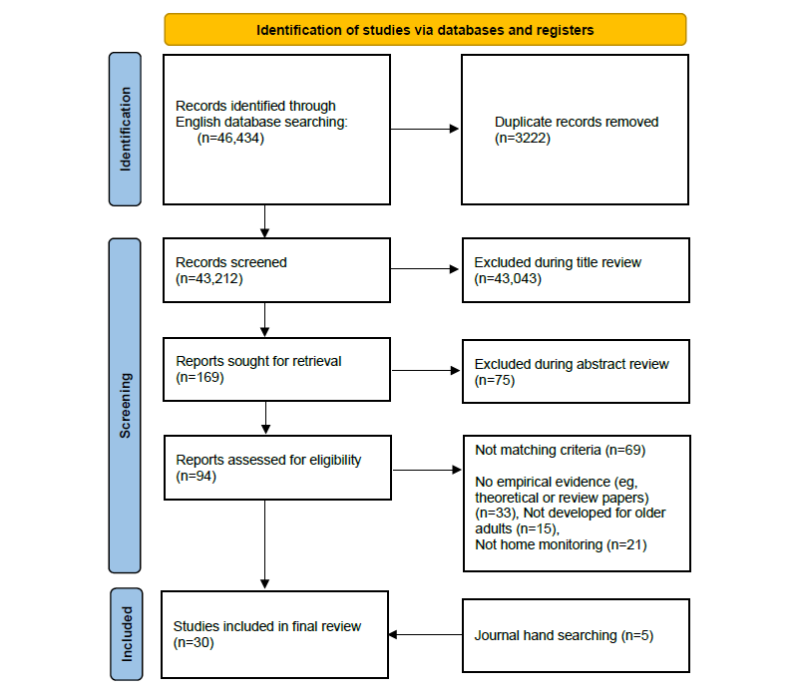
Flow diagram for the systematic scoping review.

As illustrated in the PRISMA flow diagram in Figure 1, the literature search found 43,212 titles on four databases after removing 3222 duplicates; 94 papers remained after titles and abstracts were reviewed, and those that were not germane to the study were eliminated. Subsequently, the 94 full texts were reviewed and an additional 69 were excluded based on the inclusion and exclusion criteria. Five additional articles were found through a manual search. A total of 30 research articles were finally included and used in the review. The overview of the 30 included articles is provided in Multimedia Appendix 1.

### Features of Smart Home Technologies

First, to identify currently available in-home monitoring technology for older adults, it is important to understand the types and functions of in-home monitoring technology. Demiris et al [9] classified smart home technology types and functions for an aging society as follows: (1) physiological monitoring, involving data collection and analysis of physiological measurement data such as blood pressure and pulse; (2) functional monitoring/emergency detection and response, involving data collection and analysis of functional measurement data such as those associated with daily activities; (3) safety monitoring and assistance, involving data collection and analysis of environmental hazards such as flooding and fire; (4) security monitoring and assistance, involving data collection and analysis of human threats such as those associated with security alarms; (5) social interaction monitoring and assistance, involving data collection and analysis of social interactions such as those associated with computer (online) or phone usage; and (6) cognitive and sensory assistance, involving assistive technologies to compensate for memory deficits such as reminders or task instructions.

**Figure 2:**
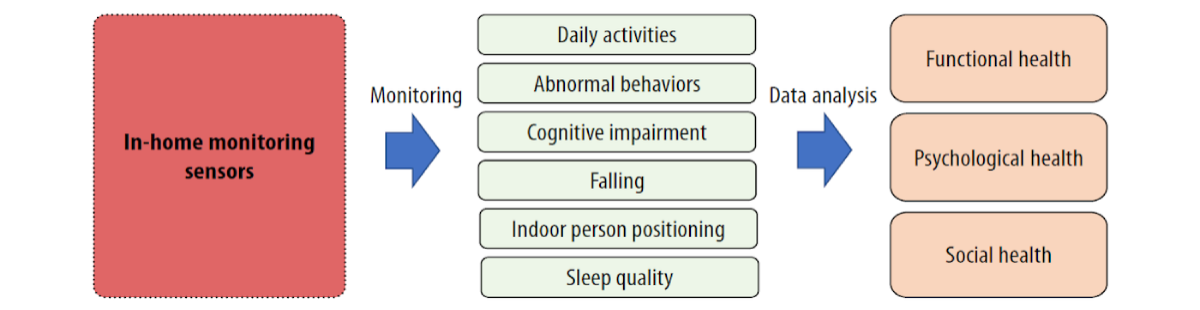
Features of smart home technologies.

All of the reports reviewed (N=30) discussed how in-home monitoring technologies can be used to monitor older adults’ activities, as well as how to analyze and detect daily activity anomalies through machine-learning algorithms. Most of the research (n=27) focused on functional monitoring and analysis, such as activities of daily living (ADLs), instrumental activities of daily living (IADLs), or falls among older adults; a few (n=3) covered social interaction monitoring. This scoping review found six functions of in-home monitoring technology among the reviewed papers: daily activities, abnormal behaviors, cognitive impairment, falls, indoor person positioning, and sleep quality (Figure 2).

### Monitoring Daily Activities

Six of the examined investigations monitored and observed older adults’ daily activities in their homes using sensor technologies, and demonstrated the feasibility and reliability of ambient home-sensing platforms in older adults’ residences [10-15]. One case study reviewed had installed sensors in older adults’ homes and analyzed the participants’ behaviors (eg, sleeping, cooking, water usage) by advanced models and algorithms for 3 months. The smartphone and computer application system used was capable of reminding the older residents to take their medications if they forgot. A contact sensor installed near the medicine box made this possible [14]. Another reviewed study monitored older adults’ activities in their homes and asked them to confirm their activities using a mobile app to validate sensor data analysis [11]. The findings showed that sensor data accurately matched the self-survey.

### Monitoring Abnormal Behaviors

Seven studies were included that identified older adults’ functional, psychological, and social abnormal activities such as unexpected and irregular behaviors using in-home monitoring technologies and machine-learning algorithms [16-22]. For example, movement at 3 AM in a living room can be considered abnormal behavior if monitoring data showed that older residents usually wake up at 6 AM every morning.

Two studies examined a unique methodology to detect the functional health decline of older adults using a public data set from smart home testbeds at the Center for Advanced Studies in Adaptive Systems (CASAS) [19,22]. The machine-learning algorithms developed in these studies successfully detected deviations from the long-term activity patterns of older adults living alone, and demonstrated the feasibility of detecting functional decline, singular deviation, and slow-deviating trends away from a previous activity routine. Another study detected anomaly behavior from learned ADL patterns and sent a push-button indicator to older adults when abnormal behaviors were detected. The investigation results validated the success of the model [21]. This type of in-home monitoring technology was also shown to be beneficial to caregivers caring for older adults who are living alone [20]. The home care monitoring system warned caregivers if an unusual behavior was detected based on learned behavioral patterns (eg, no motion was detected in the living room until 8:30 AM).

The common assumption in abnormal behavior detection research is that older adults follow regular routines in their homes and that irregularities are abnormal. However, routine-based anomaly detection fails if monitored older adults have irregular daily routines. To address this issue, another research team [17] assumed that illness can cause changes in activity levels, time, and locations of older adults. For example, those who feel unwell may spend an unusual amount of time watching television or they may walk more slowly than normal. Research findings have shown that heatmap analysis effectively detects unusual behavior determined by activity trajectories, and that it successfully distinguishes between normal and abnormal days with 88% accuracy [17].

Previous research has demonstrated the possibility of assessing psychological and social health conditions such as depression, emotional states, and even loneliness through a unique data analysis using sensor data of older adults’ home activities. In-home monitoring sensors were used to measure daily out-of-home hours, number of phone calls, computer use, walking speed, and mobility, and loneliness was also assessed during four separate periods [16]. The investigators found a significant relationship between predicted loneliness and observed loneliness, demonstrating that it is possible to estimate older adults’ loneliness by analyzing sensor data. Other studies [18] have estimated older adults’ depression levels by analyzing daily activities, demonstrating that proposed algorithms can effectively detect depression with up to 96% accuracy.

### Monitoring Cognitive Impairment

Many investigations have introduced unique sensor technology and machine-learning algorithms to detect older adults’ cognitive impairments by analyzing daily activities at home [23-27]. Researchers at an ambient assisted living lab invited two older adult groups—healthy older adults and older adults with mild cognitive impairment (MCI)—to participate in the study and monitored their behaviors [26]. Participants were asked to complete five tasks in different areas of their homes; sensor technology measured the total amount of time used to perform each task. Sensor-based observation showed that the MCI group spent more time in the kitchen looking into the refrigerator and cabinets than the healthy group. This observation provides a possible means for detecting age-related cognitive decline by monitoring performance and comparing it to IADLs. Various machine-learning models have been developed to identify older adults’ cognitive decline by utilizing a public data set from CASAS and the Oregon Center for Aging and Technology (ORCATECH) [23,25,27]. Employing various statistical models, the developers demonstrated the possibility of detecting cognitive impairments and identifying early signs of dementia with a high degree of accuracy using in-home behavior data.

Other researchers [24] developed simple nonpharmacological interventions for older adults with MCI to address abnormal behaviors related to problems such as sleep disturbance and medication interference, and demonstrated the usability and effectiveness of monitoring technology. For example, a 12-week pilot study with five older adults with dementia introduced sensor-based technology designed to guide or redirect their participants prone to nighttime wandering toward the bedroom or bathroom using smart lighting and speakers. The results demonstrated that the technology employed improved the nighttime safety of people with dementia, and also showed that depression and anxiety among caregivers had been significantly reduced.

### Monitoring Falling

In-home monitoring technology has also been designed to detect older adults’ falls in their homes [28-35]. We found two types of fall detection technology: vision-based and nonvision-based technologies.

Lotfe et al [32] utilized computer vision–based fall detection technology with a Kinect sensor, and found the approach to be highly reliable and accurate after analyzing recorded images of falls taken by older adults and their daily activities [32]. Other researchers developed a low-cost fall detector system composed of a camera and artificial vision algorithms, which also detected falls with a high degree of accuracy (>96%) [33]. Yet another study employed a Kinect sensor that effectively identifies the human skeleton, which can be used to monitor the gestures and posture of the human body [35]. The researchers placed two Kinect sensors in a bedroom and recorded various video images, including falling. The proposed algorithms were highly accurate in detecting falls and could predict falling risk after analyzing the posture and movement of a participant.

In addition to the aforementioned image-based falling detection strategies, many investigations have explored nonimage-based means to accomplish the same goal while addressing privacy concerns. Since older adults’ gait levels are significantly related to fall risk, Muleidat and Tawalbeh [31] developed a 128-sensor “smart carpet” to monitor gait parameters and detect falls. They demonstrated that their algorithm can successfully detect older adults’ falling risk as well as monitor functional decline in real time. Recently, radar-based human motion–detecting technologies have gained the attention of researchers owing to their capacity to detect postures off micro-Doppler signatures reflected by the human body. Utilizing this technology, a fall detection system was developed based on a millimeter-wave radar sensor used in conjunction with the line kernel convolutional neural network deep-learning model [30]. The system could identify a falling sequence using passive baseband data collected directly by the radar sensor with 98.74% accuracy and an average prediction time of 51.4 milliseconds. Similarly, Ding et al [28] proposed a fall detection method based on a millimeter-wave radar sensor with a k-nearest neighbor algorithm, demonstrating a high (90.83%) accuracy rate.

Fall detection systems that utilize existing in-home infrastructures such as WiFi networks have been reported. Hu et al [29] proposed an environment-independent passive fall detection system called “DeFall.” Their strategy was to analyze WiFi signal interference patterns created when a falling motion occurs. After extracting such signal patterns from the channel state information of the WiFi packets, augmented dynamic time warping algorithms were embedded to identify the acceleration patterns for a typical human fall. Their prototype achieved a detection rate of 95% in either a line-of-sight or a nonline-of-sight case using only a couple of WiFi transceivers. In addition, a few studies have focused on specific risks such as staircase and bed fall risks and detections. A bed monitoring system was developed based on infrared and pressure sensors that were preinstalled on the bed frame [34]. The sensor system successfully detected specific transitions of five typical movements associated with bed use at home (lying, sitting, standing, and exiting), and was shown to be highly effective with a detection accuracy rate of nearly 90%.

### Indoor Person Localization

One essential feature of in-home monitoring is the ability to locate the end user in a real-time manner [36,37]. In most scenarios, especially in digital health settings, location information helps the ADL recognition system infer a human subject’s activity based on their most recent activity. Additionally, based on the user’s current location, this feature enables the entire smart ecosystem to react and provide corresponding services that are available within the setting. Studies have utilized numerous technologies to provide an indoor localization service.

A multisensor human subject localization solution was proposed to incorporate various combinations of sensing devices, including radiofrequency identification transceivers, infrared, touch, and light sensors, that are mounted on furniture and appliances in the environment [36]. This unobstructed system is leveraged by adopting probabilistic models such as Bayesian network and k-nearest neighbors to infer the likelihood of the human subject’s presence within the monitoring space given the context values provided by the sensory combinations. Experimental results have shown that the system can achieve submeter localization accuracy and further provide indoor tracking capability. Alternatively, as an extended feature of the smart carpet approach mentioned above, studies have also been utilizing the concept of “smart floors,” which are embedded with pressure-sensing devices to perform indoor localization and tracking. Lan et al [37] recently proposed implementing such a feature with advanced pressure-sensing technologies and they constructed a complete smart floor prototype for indoor localization demonstration. The system was modeled with a Bayesian inference–based decoding scheme and could perform mutisubject real-time localization under submeter accuracy.

### Monitoring Sleep Quality

One reviewed study assessed and predicted older adults’ sleep quality using behavioral data. Since sleep quality plays an essential role in older adults’ health and well-being, early sleep disorder detection can be very important when addressing sleep-related issues. It has been demonstrated that a simple sensing technology and data analysis algorithm can effectively monitor older adults’ sleep-wake conditions and assess sleep quality [38]. The proposed sensing environment could monitor sleep duration, sleep latency, and awakenings during the night, and found a strong correlation between surveyed data about older adults’ sleep quality provided by social workers and the investigators’ proposed sleep quality data.

### Sensor Data and Sensor Locations

This review identified 16 types of sensor technologies and the specific location of each technology. Table 1 provides an overview of the sensor data and sensor locations.

**Table 1:** Overview of sensor data and locations.

Sensors	Number of articles	Data	Location
PIR^a^ motion sensor	21	Duration of specific activities and walk speed	Room areas (ceiling or walls of rooms)
Contact sensor	19	Occupancy of rooms or usage of items	Doors/windows, drawers, cabinets, medical boxes, refrigerators, etc
Pressure sensor	13	Duration of specific activities (eg, sleeping, resting)	Chairs, couches, floor, and beds
Light sensor	11	Lighting usage	Room areas (walls of rooms)
Temperature sensor	9	Indoor temperature	Bedroom, living room, kitchen
Electric sensor	9	Electricity usage, use of electrical appliances	Televisions, heaters, air conditioners, and lights/switchboard
Water sensor	6	Water usage	Kitchen and bathroom (water channels, kitchen floors, toilet bowls, sinks, and bathtubs)
Humidity sensor	5	Indoor humidity	Rooms (close to air conditioners)
Depth camera	3	Body image (gestures and postures) and presence	Rooms (corners of rooms)
Kinect motion sensor/infrared camera	2	Body image (gestures and postures) and presence	Rooms (walls of rooms)
Millimeter-wave frequency modulated continuous wave radar (FMCW)	2	Body posture and motion	Rooms (walls or static furniture)
Smart carpet	2	Falling, gait, positioning, and sociability	Room areas (floor)
Computer monitor	1	Daily computer use	Computer monitoring
Phone monitor	1	Daily phone use	Each phone

^a^PIR: passive infrared.

The most common data types encountered were passive infrared (PIR) motion sensors (n=20) and contact sensors (n=19), which were used to monitor human behaviors such as bodily presence and time spent on activities. The presence of PIR motion sensor monitors in each area captured the duration of specific activities such as cooking, eating, nighttime toileting activities, and similar. Four sensors arranged in a straight line 2 feet apart also monitored older adults’ mobility [16]. They were usually installed on the walls or ceilings. The range of the sensors varied, but on average, a single detector ranged up to 30 feet.

Contact sensors, also called magnetic switch or reed sensors, use magnetic flow to monitor a particular activity such as opening and closing a door, cabinet, window, or refrigerator. A signal is generated when two magnets near each other are distanced. Sensors are placed on doors, windows, drawers, or cabinets, and they monitor room occupancy or use of items such as medical boxes and refrigerators. Both PIR motion sensors and contact sensors provide information about older adults’ specific activities within their home, allowing machine-learning analysis to better understand older adults’ physical, psychological, and social health within their homes. Pressure sensors (n=12) monitor residents’ use of specific furniture pieces such as chairs and beds by detecting transitional movement. Sensors discussed in the reports were installed under beds, chairs, or couches to monitor specific behaviors such as sleeping and sitting.

Many investigations also used a variety of environmental sensors to detect and measure light (n=10), temperature (n=9), electricity (n=9), water (n=6), and humidity (n=5). For example, light sensors captured lighting usage and were installed in each room. Electric sensors connected to electrical outlets were used to monitor electricity consumption and electrical appliance usage such as televisions, heaters, and air conditioners. A water sensor was used to measure the frequency of water usage in a kitchen and bathroom water channel.

Depth cameras (n=3), Kinect motion sensors (n=2), and radar sensors (n=2) installed on a wall or corner of each room were also used to capture specific body images such as gestures and postures. Depth cameras have several advantages compared to PIR motion sensors when monitoring older adults’ activities. Since a depth camera can detect specific information such as movement, it can handle the presence of several individuals within a space, whereas a motion sensor can detect only one individual at a time. In addition, depth cameras can distinguish animals from humans. Depth camera use for human motion analysis has become an active area of research; it can recognize particular actions, falls, and other useful behaviors. Similarly, a radar sensor can be used to detect falls and can be attached to an indoor wall or piece of static furniture at a height of around 1.5 meters. One study used computer and phone monitor sensors to monitor daily computer and phone use; the information was then used to understand the social health of older adults [16]. A smart carpet was introduced to monitor older adults’ gait levels and falling episodes [31].

## Discussion

### Principal Findings

The aim of this investigation was to identify recent smart home technologies that monitor older adults’ health and to architecturally describe these technologies as they are used in older adults’ homes. This scoping review identified 30 studies focusing on in-home monitoring technology for older adults and identified key factors: types and functions of in-home monitoring technologies, the usefulness of in-home monitoring technologies, types of activity and behavior monitored by in-home monitoring technologies, and specific locations of sensing technologies. Here, we aim to extend the research findings into the implications of the identified in-home monitoring technologies to older adults’ homes.

First, this review found that in-home monitoring technologies can play a pivotal role in establishing successful aging in place, and that these technologies should be considered when developing home modification plans for older adults. Understanding an older adult’s daily activities at home is particularly critical for aging in place because changes in health status are usually determined through changes in ADLs or IADLs. Health assessments are important for older adults because they provide a basis for health professionals and caregivers to address unique health-related needs. Although health assessments generally need to be provided by highly skilled professionals, this study shows that many assessments have been accomplished via older adults’ self-reports or those of their caregivers. Nevertheless, accurately measuring older adults’ functional, psychological, and social health conditions through a self-survey has limitations [39], especially those completed by older adults with MCI.

In-home monitoring technology can be beneficial to both older adults needing help with daily living and their care providers. Various sensor technologies are capable of monitoring older adults’ activities with a high degree of accuracy and reliability. Furthermore, the collected sensor data provide a machine-learning analysis that can offer a comprehensive overview of the older adult’s health condition and ability to function independently within the home. Sensor technologies and unique data analysis methods can automatically identify older adults’ hidden wellness parameters such as abnormal behavior, cognitive impairment, falling, and sleep quality. Health abnormality identification is of paramount importance in that the early identification of change enables older adults to receive early intervention that can play a pivotal role in minimizing the risk of further health decline. Much of the published research reviewed for this study demonstrated that in-home monitoring technologies can predict risks to older adults’ physical, cognitive, psychological, and social health.

One of the critical issues in aging in place is the lack of formal support caused by the current professional caregiver shortage. Family members who bear the heavy burden of care for older adults often experience physical, emotional, social, and financial challenges. It is difficult to care for an older adult who has a cognitive impairment, especially those who wander at night, which is a major cause of caregiver burnout. Sensor-based technology has the capacity to monitor older adults’ daily activities, falls, nocturnal restlessness, and eating behavior, thereby reducing caretakers’ burden of care as well as accompanying stress and anxiety [40].

Second, it is essential to help older adults accept the aforementioned technologies’ presence in their homes through appropriate design strategies. According to previous reviews, older adults’ perceptions of usefulness, ease of use, privacy, and affordability are significantly related to their attitude and intention to use smart home products [41]. There are two implementations of in-home monitoring technologies: (1) sensors worn by older adults and (2) sensors embedded in the built environment (eg, wall, door) or attached to products such as furniture and kitchen appliances. To support ease of use, in-home monitoring technology should be developed as an ambient sensor system located in stationary home areas. Even though individual wearable sensors are commonly utilized in smart home technology, some older adults are reluctant to use them because they must constantly be worn somewhere on the body to be useful and the battery status must be checked on a regular basis. This is a particularly negative factor among older adults with MCI.

As described in Table 1, in-home technologies can be implemented into each area of senior housing for a specific purpose. For example, PIR motion sensors can be installed in room areas to monitor older adults’ activities. Previous research highlighted that most older adults are positively motivated to use a sensor monitoring system; however, privacy is a critical concern for many [39]. How aware individuals are of a sensor being present in their immediate environment varies from one user to another. Some older residents quickly forget about their monitoring equipment, while others consider sensors irritating and therefore change their behavior or activity routines. For example, contact sensors attached between a door and door frame are readily noticeable and bother some residents. PIR motion sensors are usually installed high on a wall corner in a high-traffic area for a wide view. Some older adults may feel uncomfortable because they feel as if someone is observing them. Moreover, monitoring devices might be considered a stigma or a symbol of dependence. Thus, it is critical to address both privacy concerns and make sensor technology less obvious when incorporating in-home technologies into the home environment. That is, creative design solutions are needed to address these issues. An ambient sensor system should be placed above the typical human field of view, and a concealed sensor system would help address the privacy issue. For example, magnets used in contact sensors can be concealed within the door frame, but they should be easily accessible for maintenance. Smart home technologies should be considered during the early stages of home design or home modification plans instead of adding them after the design or construction process is complete.

### Limitations

The aim of this investigation was to identify recent in-home monitoring technologies, which can be applied in older adults’ homes. To ensure that the research reports were up to date, the publishing span was limited to articles bearing dates between June 2016 and 2021. Furthermore, this research aimed to identify in-home monitoring technology that can be applied in older adults’ homes. However, since there is no clear definition of in-home monitoring technology, the review included sensing technology designed to monitor or track human behavior or activity. Our search string included two different terms used in previous research, although other terms may also be available. Thus, this process may not have found all of the research on in-home monitoring technology for older adults. This review included only studies written in English. Therefore, we may have missed other articles written in other languages. After consultation among the authors, only one author (DK) analyzed the included studies. Thus, this might have introduced bias and impacted the results. However, the identified research and main key characteristics were thoroughly discussed among all authors.

Even though wearable devices offer a huge opportunity to monitor and track older adults’ activities and behavior, this review excluded wearable sensing technology because of its acceptability and feasibility issues for older adults [42]. For example, older adults with MCI may forget to wear or charge these devices.

### Conclusion

This review provides a key summarization of in-home monitoring technologies, an underexplored research area in the field of aging in place. This wide-ranging synthesis demonstrates that in-home monitoring technologies within older adults’ homes play an essential role in aging in place, in that the technology monitors older adults’ daily activities and identifies various health-related issues. These findings will be beneficial for architects and designers when developing home modification strategies or senior housing for successful aging in place, in that this research provides key characteristics of in-home monitoring technologies for older adults residing in their own homes. Utilizing this technology, older adults can be more proactive in terms of their health rather than reactive; they can receive early intervention after early identification. This research also highlights the importance of appropriate strategies for smart home design. The emergence of telehealth and online communication can play an essential role in successful aging in place, in that this telemedicine is an effective way to address many societal issues of older adults, such as overcrowding, driving a car, and mitigating infection risks [43]. A smart home with in-home monitoring technology can provide significant information to care providers, which will be helpful for maintaining health and well-being.

## Appendix

Multimedia Appendix 1 Overview of 30 included studies.

Multimedia Appendix 2 PRISMA-ScR checklist. PRISMA-ScR: Preferred Reporting Items for Systematic Reviews and Meta-Analyses extension for Scoping Reviews.

## Abbreviations

ADL: activity of daily living
CASAS: Center for Advanced Studies in Adaptive Systems
IADL: instrumental activity of daily living
MCI: mild cognitive impairment
ORCATECH: Oregon Center for Aging and Technology
PIR: passive infrared
PRISMA-ScR: Preferred Reporting Items of Systematic Reviews and Meta-Analysis extension for Scoping Reviews
