# Supplementary material for: In-Home Monitoring Technology for Aging in Place: Scoping Review
Source: Interact J Med Res. 2022 Sep 1;11(2):e39005. doi: 10.2196/39005 (PMC9478817; doi:10.2196/39005)
Supplement: Multimedia Appendix 1 [file ijmr_v11i2e39005_app1.docx]

| **Author (year)** | **Purpose** | **Sample** | **Setting** | **Technology** | **Technology type & behavior monitored** | **Outcome** |
| --- | --- | --- | --- | --- | --- | --- |
| Austin et al., (2016) | To estimate loneliness in older adults using an array of in-home sensors | 16 older adults | Home  (8 months) | Motion sensors, contact sensors, phone monitor, computer | Soc: Loneliness | This research demonstrated a comprehensive sensor system to assess loneliness in older adults using an in-home assessment process. |
| Lu et al., (2016) | To present a in-bed monitoring system to distinguish the transition of typical movements around home-use beds, thereby to prevent falling hazards | 5 male volunteers (age 21-27) | Home, stainless steel bolding bed (length:180cm, width: 120cm, height: 30cm) | Infrared sensors, pressure sensors | Fx: In-bed falling | The experiments demonstrated that the system is highly effective with an accuracy of nearly 90% detection rate of bed falling motions. |
| Ruan et al., (2016) | To develop a real-time indoor personnel localization system based on RFID and hybrid sensor combinations | 3 volunteers | Lab | RFID transceivers, infrared, touch, light sensors | Fx: Indoor personnel positioning | The system overall achieves a sub-meter indoor human localization and tracking, which provides sufficient positioning capability in an indoor setting. |
| Kasteren et al., (2017) | To explore the usefulness of sensor technologies capturing daily routines of older adults | 3 older adults | Home  (6 months) | Motion sensors, contact sensors, acoustic sensor, environmental sensors, electronic thermometer, electronic scales, circuit meter, accelerometer | Fx: Daily activities | The sensor data identified older adults' behavioral patterns in their homes and accurately matched self-reported routines. |
| Vildjiounaite (2017) | To detect abnormal behaviors using depth sensor-based detection | 4 older adults | Home  (40-78 days) | Depth camera | Fx: Daily activity & abnormal behavior | The proposed sensor system detector distinguished between older residents' behavior when they are normal and sick. The average accuracy was 88%. |
| Kim et al., (2017) | To develop a simple and unobtrusive sensing system that can effectively monitor and detect depression levels in elderly who are living alone. | 20 older adults | Home  (3 months) | Motion sensor, gateway, bed sensor, contact sensors | Fx & Soc: Daily activities & depression | The daily activities data from the sensing system show a high correlation with the depression level of the elderly from the survey. The sensing system can be used for monitoring the elderly’s mental health. |
| Mongkolnam et al., (2017) | To detect and clarify human gestures and postures using Kinect | 5 volunteers | Lab | Kinect sensors | Fx: Falling: gestures and postures (skeletons) | This research leverages the Kinect’s infrared sensing capability, which can effectively identify a human skeleton both in daytime and nighttime. |
| Miguel et al (2017) | To present a new low-cost fall detector for smart homes based on artificial vision algorithms | 53 videos were recorded in two different locations (lab and home) | Lab & Home | Fall detection system (camera, Raspberry Pi, CPU) | Fx: Falling | This research was conducted on over 50 different fall videos analyses and results have shown a detection ratio of greater than 96%. |
| Akl et al (2017) | To develop a statistical model detecting mild cognitive impairment of older adults through home monitoring | 68 older adults from ORCATECH^*^ | Home  (3 years) | Motion sensors | Fx: Daily activities & early signs of dementia | The proposed model demonstrated that mild cognitive impairment of older adults can be detected after analyzing their daily activities. |
| Lan et al., (2017) | To develop a pressure-sensing-based smart floor for indoor personal localization | Multiple lab staff | Lab  (3.6m by 3.6M testing space) | Pressure sensors | Fx: Indoor personnel positioning | The developed prototype demonstrated the capability of multi-subject real-time localization under an unobstructive setting with sub-meter level accuracy. |
| Caroux et al., (2018) | To assess the robustness and reliability of activity verification and context-aware assistive applications for aging in place | 7 older adults | Home  (8 weeks) | Motion sensor, contact sensors, and smart switches | Fx: Daily activity | Results showed a high level of applicability to context-aware assistive applications for aging in place. Its inter-participant and intra-participant consistencies were demonstrated. |
| Aramendi et al., (2018) | To unobtrusively identify functional health decline of older adults | 29 older adults from CASAS smart home testbeds^***^ | Home  (19 months) | Motion sensor, contact sensors, light sensor, item sensors, temperature sensor | Fx: Daily activities & functional health decline | This method demonstrated the possibility of identifying older adults' functional health decline unobtrusively in their home using behavior data. |
| Lotfi et al., (2018) | To propose a computer vision-based fall detection approach suitable for a home environment through analyzing the motion and shape of the human body | 70 videos from UR Fall detection dataset^**^ | Lab | Kinect sensors, accelerometer | Fx: Falling | Experimental results show the reliability and the robustness of the proposed approach with a high fall detection rate of 99:60% and a low false alarm rate of 2:62% when tested with the UR Fall Detection dataset. |
| Alberdi et al., (2018) | To detect changes in psychological, cognitive, and behavioral symptoms of AD using smart home behavior data and machine learning techniques | 29 older adults from CASAS smart home testbeds^***^ | Home  (+2 years) | Motion sensor, contact sensor, pressure sensor, water flow sensor | Fx: Daily activities & early sign of dementia | This work has demonstrated the possibility of predicting mobility, cognitive, and mood-related symptoms from unobtrusively collected in-home behavior data. |
| Yu et al (2019) | To monitor older adults' daily activities in a residential environment using unobtrusive sensors | One older adult | Home  (3 months) | Temperature and humidity sensory, switch sensor, pressure sensors, water, and electricity sensor | Fx: Daily activities | Unobtrusive sensors identified older adults' daily activities without influencing their normal activities. |
| Lach et al., (2019) | To evaluate home monitoring system to track activity and sleep in community-dwelling older adults for use. | 10 older adults (women) | Home  (3 months) | Motion detectors, Pressure sensors, contact sensory | Fx: Daily activity | The use of the sensor system was feasible in this pilot study and acceptable to participants. Activity and sleep data were similar across self-survey. Participants were generally positive about the monitoring system. |
| Grguri´c et al., (2019) | To detect daily activity patterns and automatically issue a warning | 13 older adults | Home  (6 months) | Contact sensors, power sensors, motion sensors, environmental sensors (e.g., luminance, temperature, and humidity) | Fx: Daily activities & anomal behaviors | The system was successfully validated and identified 23 patterns per single household. On average, 61% of proposed rules were accepted by the caregivers |
| Ghayvat et al., (2019) | To propose an anomaly detection method based on a sensing system | 4 older adults | Home  (300 days) | Pressure sensor, temperature sensor, motion sensor, smoke detector, electronic and electrical appliance usage monitoring sensor, contact sensor | Fx: Daily activities & anomaly behaviors | The new feature set from sensor data enhances the system accuracy up to 98.17% and demonstrated a high validity. |
| Susnea et al., (2019) | To propose a method to monitor the activity of the elderly living alone and detect deviations from the previous activity patterns | Two older adults from CASAS smart home testbeds^***^ | Home  (1 month) | Motion sensor, contact sensor, pressure sensor, water flow sensor | Fx: Daily activities & abnomal behaviors | The proposed method successfully detects both singular deviations and slow-deviating trends from the previous activity routine of the monitored persons. |
| Lazarou et al., (2019) | To investigate the long-term effects of assistive technology for people with cognitive impairment | 18 older adults with cognitive impairment | Home  (4-12 months) | Electric sensor, contact sensor,  motion sensor, pressure sensor, depth camera | Fx: Daily activities & cognitive impairment | Participants, who received the sensor-based system, have shown improvement in domains such as sleep quality and daily activity, as measured by the multi-sensory system. |
| Wang et al (2020) | To visualize and examine residents' status and the daily activities of older adults in their homes | 16 older adults | Home  (6-36 months) | Motion sensors, contact sensors, power sensors, water sensors, and various environmental sensors (e.g., track changes, in temperature, luminance, and humidity). | Fx: Daily activities | This ambient sensor network captures older adults' daily activities and patterns with high accuracy rates. |
| VandeWeerd et al (2020) | To describe a comprehensive ambient home-sensing platform for older adults | 21 older adults | Home  (6 months) | Contact sensors, motion sensors, energy sensors, pressure sensors, water sensors, and environmental sensors | Fx: Daily activities | The ambient sensing platform offers the potential to monitor older adults within their own homes, facilitating supportive environments that bolster the healthy, safe, and independent aging plan preferred by older cohorts. |
| Muheidat & Tawalbeh (2020) | To develop a smart carpet consisting of a sensor pad placed under a carpet for monitoring walking activity and falling | 10 volunteers | Lab | Smart carpet | Fx: Falling & gait | This research demonstrated that the proposed smart carpet detects falls with 95% sensitivity and measuring and estimating gait can be used to predict a falling risk and social activity. |
| Lazarou et al., (2020) | To determine smart technologies to (re)direct a PWD that is wandering at night and reduce caregiver's burden. | 5 older adults with cognitive impairment | Home  (3 months) | Motion sensor, contact sensor, pressure sensor, speaker, and smart bulbs | Fx: Wandering at a night | The average depression and anxiety in caregivers have been reduced after the 12weeks. The proposed system has proven successful in supporting the safety of persons with dementia as well as their caregivers. |
| Ahamed et al (2020) | To identify the early signs of dementia of older adults through smart home technologies | 304 residents from CASAS smart home testbeds^***^ | Home  (+2 years) | Motion sensor, door sensor, light sensor, contract sensor, temperature sensor | Fx: Daily activities & early signs of dementia | The machine learning process identified the cognitive status of monitored adults and detected the early sign of dementia with 90.74% accuracy. |
| Lussier et al., (2020) | To identify difficulties of IADL of mild cognitive impairment (MCI) in older adults using sensor-based data | 48 older adults | Lab | Motion sensor, electric sensor, contact sensor | Fx: Daily activities & early signs of dementia | Sensor-based observation showed that MCI group spent more time in the kitchen and looking into the fridge and kitchen cabinets than CH participants. This research found the potential for the detection of MCI in older adults when they perform IADL. |
| Wang et al., (2020) | To develop a non-image-based, privacy conservative falling detection system with fewer network parameters and less computation cost | 11 young (age 22-28) volunteers | Lab | Millimeter-wave frequency modulated continuous wave radar (FMCW) | Fx: Falling | The system is able to identify the falling sequence with the passive baseband data collected directly by the radar sensor with an accuracy of 98.74% and an average prediction time of 51.4 milliseconds. |
| Hu et al., (2020) | To propose an environment independent passive fall detection system that is based on existing in-house WiFi infrastructures | 2 volunteers, 1 human-like dummy | Lab  (3 months) | WiFi transceivers | Fx: Falling | The prototype can achieve a human fall detection with 95% accuracy in either line-of-sight (LOS) or non-line-of-sight (NLOS) case using only a pair of WiFi transceivers. |
| Kim et al., 2021 | To propose a simple and affordable unobtrusive sensing environment for monitoring the elderly’s sleep-wake conditions, to assess their sleep quality | 4 older adults | Home  (2 months) | Motion sensory, pressure sensor, contact sensor | Fx: Sleep-wake condition (sleep quality) | This sensor system can effectively detect sleep quality based on a simple unobtrusive sensing system. |
| Ding et al., (2021) | To propose a non-image-based falling detection method by tracking elderly’s walking velocity and acceleration | 5 volunteers | Lab | Millimeter-wave frequency modulated continuous wave radar (FMCW) | Fx: Falling | The experiment shows that given the experimental setup, the falling motion samples within a total of 300 samples can be identified with an accuracy of 91.25% |

Multimedia Appendix 1: Overview of 30 included studies

* Oregon center for aging and technology (ORCATECH) is a multi-disciplinary organization that is transforming clinical research by developing and implementing leading-edge technologies that measure life's data in real-time.

** UR Fall Detection Dataset at the University of Rzeszow provides 70 (30 falls + 40 activities of daily living) sequences recorded by two Microsoft Kinect cameras and corresponding accelerometric data.

*** CASAS smart home testbeds is a multi-disciplinary research project at Washington State University focused on the creation of an intelligent home environment.

CASAS dataset is a widely used dataset that has been developed by Washington State University.
